# Occurrence, Toxicity, and Analysis of Major Mycotoxins in Food

**DOI:** 10.3390/ijerph14060632

**Published:** 2017-06-13

**Authors:** Ahmad Alshannaq, Jae-Hyuk Yu

**Affiliations:** 1 Department of Food Science, University of Wisconsin-Madison, 1550 Linden Drive, Madison, WI 53706, USA; alshannaq@wisc.edu; 2 Food Research Institute, University of Wisconsin-Madison, 1550 Linden Drive, Madison, WI 53706, USA; 3 Department of Bacteriology, University of Wisconsin-Madison, 1550 Linden Drive, Madison, WI 53706, USA

**Keywords:** fungi, mycotoxins, aflatoxin, toxicology, analysis, chromatography, rapid strip test

## Abstract

Mycotoxins are toxic secondary metabolites produced by certain filamentous fungi (molds). These low molecular weight compounds (usually less than 1000 Daltons) are naturally occurring and practically unavoidable. They can enter our food chain either directly from plant-based food components contaminated with mycotoxins or by indirect contamination from the growth of toxigenic fungi on food. Mycotoxins can accumulate in maturing corn, cereals, soybeans, sorghum, peanuts, and other food and feed crops in the field and in grain during transportation. Consumption of mycotoxin-contaminated food or feed can cause acute or chronic toxicity in human and animals. In addition to concerns over adverse effects from direct consumption of mycotoxin-contaminated foods and feeds, there is also public health concern over the potential ingestion of animal-derived food products, such as meat, milk, or eggs, containing residues or metabolites of mycotoxins. Members of three fungal genera, *Aspergillus*, *Fusarium*, and *Penicillium*, are the major mycotoxin producers. While over 300 mycotoxins have been identified, six (aflatoxins, trichothecenes, zearalenone, fumonisins, ochratoxins, and patulin) are regularly found in food, posing unpredictable and ongoing food safety problems worldwide. This review summarizes the toxicity of the six mycotoxins, foods commonly contaminated by one or more of them, and the current methods for detection and analysis of these mycotoxins.

## 1. Introduction

Mycotoxins are poisonous (toxic) secondary metabolites produced by many filamentous fungi belonging to the phylum Ascomycota. Bennett [1] suggested a definition of mycotoxins as “natural products produced by fungi that evoke a toxic response when introduced in low concentration to higher vertebrates and other animals by a natural route.” Some mycotoxins can have additional effects such as phytotoxicity or antimicrobial activity. Generally, mycotoxins exclude substances such as those referred to by Bennett [1] as “mushroom and yeast poisons”. The major fungi causing frequent and problematic contamination of foods and feeds with mycotoxins are members of the fungal genera *Aspergillus*, *Fusarium*, and *Penicillium* [2,3]. While *Aspergillus* and *Penicillium* species frequently grow on foods and feeds under storage conditions, *Fusarium* species often infect growing crops such as wheat, barley and corn in the field and propagate in the plant [4,5]. Presently, over 300 mycotoxins have been identified and reported; however, only a few regularly contaminate food and animal feedstuffs. These are aflatoxins (AF), ochratoxins (OT), fumonisins, patulin, zearalenone (ZEA), and trichothecenes including deoxynivalenol (DON) and T-2 toxin [6,7].

Mycotoxin contamination of food is an ongoing global concern. Mycotoxin contamination is considered an unavoidable and unpredictable problem, even where good agricultural, storage, and processing practices are implemented, posing a difficult challenge to food safety. Additionally, many mycotoxins are not easily eliminated during food processing because of their stability against heat, physical, and chemical treatments [3,8]. Furthermore, feed contamination can also pose an extra hazard for food safety due to the possible carry-over of mycotoxins to animal-derived products such as milk, meat, and egg, leading to mycotoxin intake by humans [9]. Many strategies have been proposed for controlling the mycotoxin occurrence in different food commodities; however, no clear-cut solutions exist.

Mycotoxins threaten human and animal health, hamper international trading, waste foods and feeds, and divert resources towards research, enforcement, regulation, and applications to alleviate mycotoxin problems [10,11]. Unfortunately, about 25% of the world’s harvested crops are contaminated by mycotoxins each year, leading to huge agricultural and industrial losses in the billions of dollars [3]. Among the mycotoxins, aflatoxins (AFs) are considered the most toxic, with significant economic burden to agriculture [12,13]. In the United States (US) and European Union (EU) countries, AFs are primarily an economic concern, whereas in the developing countries of Asia and Africa, AFs contribute to hundreds of hepatocellular carcinoma cases each year [12,14,15]. Importantly, the estimated annual losses to the US corn industry due to aflatoxin contamination range from US $52.1 million to US $1.68 billion [12]. Additionally, mycotoxins are the main hazard cited in EU border rejection notifications according to Rapid Alert System for Food and Feed (RASFF), with AFs the specific mycotoxins most commonly associated with the notifications [3].

Since the initial discovery of mycotoxins, many methods have been validated and used for the analysis of mycotoxins in food and feed such as thin layer chromatography (TLC); high performance liquid chromatography (HPLC) coupled with FLD, UV, DAD or MS detection; gas chromatography (GC) coupled with ECD, FID or MS detection; Ultra Performance Liquid Chromatography (UPLC); enzyme-linked immunosorbent assay (ELISA); and rapid strip screening tests [16,17]. Although tremendous progress has been made in this area, there are still major challenges and drawbacks to these analytical methods that need to be addressed [18]. Analytical challenges include difficulties in detecting low-level mycotoxin contamination, complex food matrices in which the mycotoxin contamination occurs necessitating complicated extraction processes, the great diversity of mycotoxin chemical structures, and the co-occurrence of mycotoxins [18,19,20]. To tackle these challenges, continuous improvements in the analytical methodology for mycotoxin analysis in a variety of food matrix are needed to support the enforcement of mycotoxin regulations, protect consumer’s health, support the agriculture industry, and facilitate international food trade [19]. This review summarizes the key mycotoxins commonly contaminating foodstuffs, their toxicity, and the key methods used for their detection and analysis in a variety of foods.

## 2. Occurrence and Toxicity of Major Mycotoxins

Mycotoxin contamination can occur pre-harvest when the crop plant is growing or post-harvest during processing, packaging, distribution, and storage of food products [7,21]. Generally, all crops and cereals that are improperly stored under feverish temperature and prompting humidity for a prolonged time can be subject to mold growth and mycotoxin contamination [5]. Maize is considered to be the crop most susceptible to mycotoxins contamination, while rice is the least [22].

Most mycotoxins are chemically and thermally stable during food processing, including cooking, boiling, baking, frying, roasting, and pasteurization. Mycotoxins can also come to the human plate via animal products such as meat, eggs, milk as the result of the animal eating contaminated feed [3,23]. Many national and international public health and governmental authorities such as the US Food and Drug Administration (FDA), World Health Organization (WHO), Food Agriculture Organization (FAO), and the European Food Safety Authority (EFSA), are paying serious attention to mycotoxin contamination in food and feed and addressed this global problem by adopting strict regulatory guidelines for major mycotoxin classes in food and feed [5]. Currently, about 100 countries have established limits on the presence of major mycotoxins in food and feed [24,25]. Table 1 lists the important toxins, main producers, and some commonly contaminated food commodities along with the US FDA and EU regulatory limits for mycotoxin levels in food and animal feed.

### 2.1. Aflatoxins

**Figure 1:**
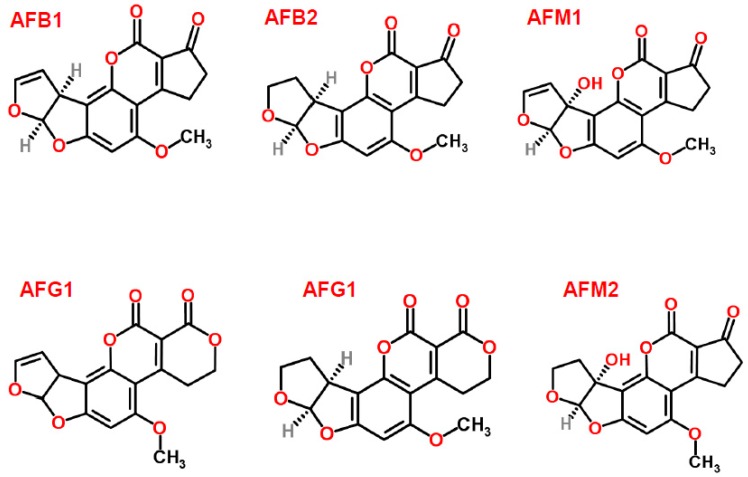
Chemical structures of aflatoxins (structures from www.chemspider.com).

Aflatoxins (Figure 1) are a group of structurally related, toxic, secondary metabolites produced mainly by *A. flavus* and *A. parasiticus* that are present normally in soil and various organic materials [14,26]. While *A*. *flavus* strains produce only aflatoxins B1 (AFB1) and B2 (AFB2), *A*. *parasiticus* strains can produce AFB1, AFB2, G1 (AFG1), and G2 (AFG2) [5]. Since the discovery of AFs as causative agents of Turkey X disease killing 100,000 young turkeys in Great Britain in 1960, AFs have been the subject of great deal of research and are considered the most studied mycotoxins [7,27].

The first outbreak of aflatoxicosis affecting humans, reported in India, led to the death of 100 people [28]. Aflatoxin-producing fungi grow on a wide variety of foods such cereals (maize, rice, barley, oats, and sorghum), peanuts, ground nuts, pistachio nuts, almonds, walnuts and cottonseeds [5,6]. Milk can be also contaminated with aflatoxin M1 (AFM1), which is a principal hydroxylated-AFB1 metabolite biotransformed by hepatic microsomal cytochrome P450 in cows fed a diet contaminated with AFB1 [5]. AFM1 can be detected in milk 12–24 h after cow consuming feed contaminated with AFB1, and the concentration of AFM1 in milk is correlated to the levels of AFB1 in the raw feedstuffs [29]. AFM1 can be detected also in some dairy products such as cheese with a concentration higher than that of the raw milk since AFM1 is heat stable, binds well to casein, and is not affected by the cheese-making process [30,31].

AFs have carcinogenic, teratogenic, hepatotoxic, mutagenic, and immunosuppressive effects, with the liver the main organ affected [5]. AFs are associated with both acute toxicity and chronic carcinogenicity in human and animal populations [5]. AFB1 is classified by the International Agency of Research on Cancer (IARC) as a Group 1 carcinogen, with high risks for hepatocellular carcinoma (HCC) in individuals exposed to aflatoxins, while AFM1 is listed in Group 2B (possibly carcinogenic to humans) [13]. Acute toxicosis, while usually rare in developed countries, is common in some developing countries, especially in Africa, whereas chronic carcinogenicity is a global problem [5,32]. The LD_50_ values range between 0.5–10 mg/kg body weight in different animal species [33]. In humans, acute aflatoxicosis is characterized by vomiting, abdominal pain, pulmonary and cerebral edema, coma, convulsions, and even death [34]. In animals, symptoms of gastrointestinal dysfunction, reduced reproduction, reduced feed conversion and efficiency, lowered milk and egg production, and anemia have been reported [35]. The toxic effects of AFB1 are principally due to the binding of bioactivated AFB_1_-8,9-epoxide to cellular macromolecules, particularly mitochondrial and nuclear nucleic acids and nucleoproteins, resulting in general cytotoxic effects [5]. Due to the extreme concerns about AF contamination in food and feed and their negative public health and economic impacts, AFs have been closely controlled by the FDA since 1969. Among all mycotoxins, AFs are the only one regulated by established FDA action levels; others are subject only to advisory levels.

### 2.2. Ochratoxins

**Figure 2:**
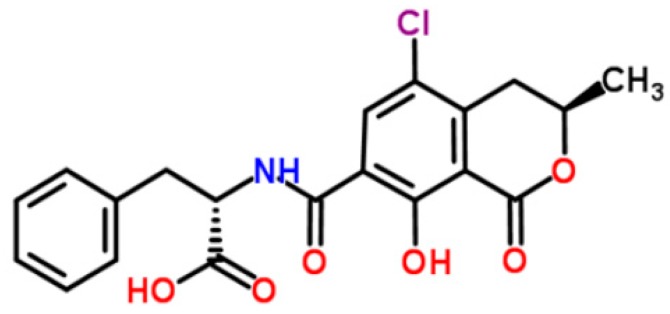
Ochratoxin A (structure from www.chemspider.com).

Discovered in 1965 in South Africa, ochratoxins are a group of related compounds produced by *Aspergillus ochraceus*, *Penicillium verrucosum*, and other *Penicillium* species. The most important toxin of the group is ochratoxin A (OTA, Figure 2) [5,36]. Generally, *P. verrucosum* can produce OTA under cool-temperate conditions, whereas *A. ochraceus* prefers to grow in hot-tropical regions [37]. Ochratoxins have been found in a wide variety of agricultural commodities such as corn, wheat, barley, flour, coffee, rice, oats, rye, beans, peas, and mixed feeds, and are notably present in wine, grape juice, and dried vine fruits [38]. Ochratoxins can also contaminate animal-derived products, such as meat and milk, and can be found in human milk [11]. Among all potential source of OTA, coffees and wines are identified as major contributors to OTA intake [13]. Importantly, OTA is very stable in acidic environments and can tolerate high thermal processing; thus, OTA can be found in cereal products, beer, and roasted coffee and is difficult to eliminate from food under normal cooking conditions [5,10].

OTA is classified by IARC in Group 2B (possible human carcinogen), and it has been suspected to cause Balkan Endemic Nephropathy (BEN: chronic tubulointerstitial disease) which affects south-eastern Europeans [13]. OTA is acutely nephrotoxic and hepatotoxic. The oral LD_50_ of OTA ranges from 3 to 20 mg/kg in different animals [8]. In addition, OTA is reported to cause immunotoxicity, genotoxicity, neurotoxicity, teratogenicity, and embryotoxicity in both human and animals [39]. OTA impacts the productivity of food producing animals by reduced feed conversion and body weight gain and may decrease egg production in laying hens [40]. As OTA is fat soluble, it tends to accumulate in the tissue of animals, especially pigs [41]. Because of its structural similarity to the essential amino acid phenylalanine, OTA interferes with phenylalanine hydroxylase activity in the kidney and liver, resulting in the inhibition of proper protein synthesis. However, OTA also inhibits RNA and DNA synthesis [42]. Until now, the US FDA has not set any regulatory guidelines for OTA. However, EU has established limits of OTA in several foodstuffs, in the ranges of 5–50 parts per billion (ppb) (Regulations (EC) No. 1881/2006) [9].

### 2.3. Zearalenone

**Figure 3:**
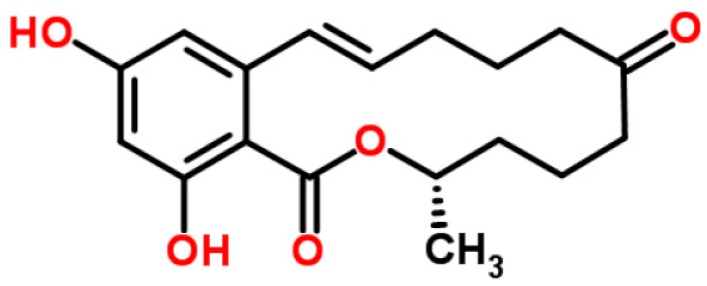
Zearalenone (structure from www.chemspider.com).

Zearalenone (ZEA, Figure 3), a macrocyclic β-resorcyclic acid lactone, is produced by *Fusarium* species, mainly *F*. *graminearum* and *F*. *semitectum* [43]. Due to its structural similarity to the naturally-occurring estrogens, ZEA is better described as an estrogenic mycotoxin that induces obvious estrogenic effects in human and animals [5]. ZEA is frequently found in corn, wheat, barley, sorghum, and rye. Corn and wheat are more frequently contaminated with ZEA in the United States and Canada, whereas, the major sources of ZEA contamination are wheat, rye, and oats in European countries [10]. ZEA production is favored by high humidity and low temperature conditions. ZEA contamination simultaneously occurs with DON, and less frequently with aflatoxins [44]. ZEA is stable under regular cooking temperatures and partially eliminated under high temperatures [45].

ZEA is classified as a Group 3 carcinogen by IARC. Public health concern over ZEA is associated with its strong estrogenic activity. ZEA binds competitively to estrogen receptors (ERα and ERβ) in a number of in vitro or in vivo models in various animal species, resulting in changes and lesions in the female reproductive system [10]. ZEA and its derivatives act by displacing estradiol from its uterine binding protein, eliciting an estrogenic response [44]. ZEA causes significant alterations in the reproductive tract of laboratory and domestic animals. Infertility, swelling of the uterus and vulva, increased embryolethal resorptions, and atrophy of ovaries have been observed in mice, rats, guinea pigs, and rabbits [46]. In cattle, consuming feed contaminated with high amount of ZEA may be directly associated with infertility, reduced milk production, and hyperestrogenism [47]. To date, there are no advisory levels of ZEA set by the US FDA. However, the European committee has regulated the maximum levels of zearalenone ranging between 20–100 ppb in various food commodities ((EC) No. 1126/2007) [9].

### 2.4. Fumonisins

**Figure 4:**
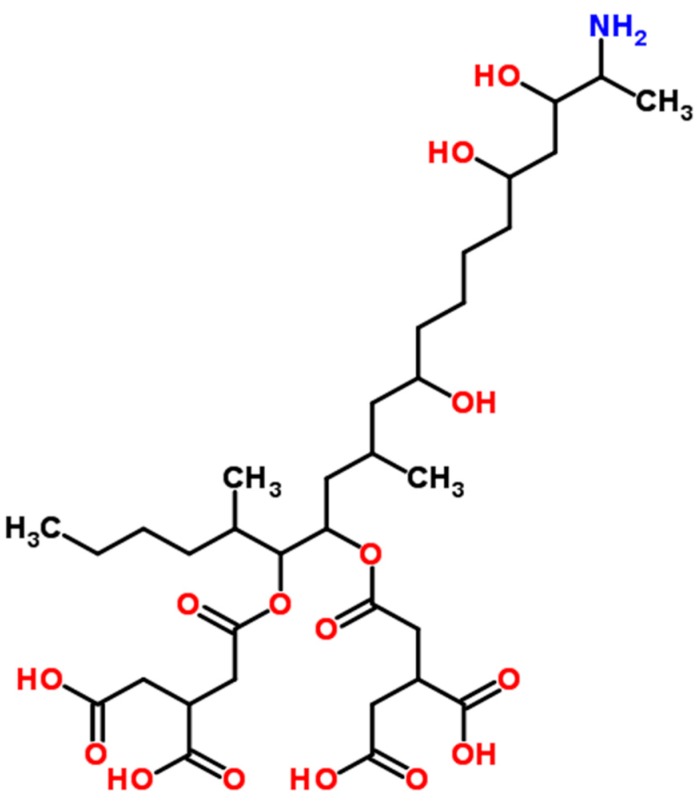
Fuminisin B1 (structure from www.chemspider.com).

Fumonisins, a group of non-fluorescent mycotoxins, were discovered in 1988 [5]. As shown in Figure 4, fumonisins are hydrophilic mycotoxins that are structurally different from most other mycotoxins, which can be dissolved completely in organic solvents. Fumonisins, produced mainly by *F. verticillioides*, were isolated from corn that was associated with the outbreak of leuko-encephalomalacia (LEM) in equine in South Africa in 1970. Fumonisins also caused pulmonary edema when contaminated corn was fed to pigs [44]. Fumonisins are also produced by *F*. *proliferatum*. Presently, over 28 fumonisins have been isolated and are classified into four groups (A, B, C and P) [48]. Fumonisin B1 (FB1) (Figure 4) is the most commonly found, comprising 70–80% of the total fumonisins family. FB1 commonly contaminates maize kernels [49]. Fumonisins can also occur in sorghum, wheat, barley, soybean, asparagus spears, figs, black tea, and medicinal plants [2,5]. In the US, *F*. *verticillioides* contaminates about 80% of all harvested corn [44]. In China, FB1, FB_2_, and FB_3_ were detected in 98.1% of corn product samples collected from Shandong Province in 2014 [50].

FB1 is the most prevalent fumonisin in human food and also the most toxic, classified in Group 2B (probably carcinogenic) by IARC [13]. Structurally, fumonisins are similar to sphinganine, and FB1 exerts its toxic effects by disrupting sphingolipid metabolism [44]. Fumonisins target mainly the liver and the kidney and cause severe toxicity in experimental animals [51]. Due to their hydrophilicity, there are no carryovers of fumonisins into milk in cattle, and little FB1 accumulates in edible tissues [9]. WHO set the provisional maximum tolerable daily intake at 2 µg/kg body weight [44]. FDA has set the recommended maximum levels at 2–4 ppm for fumonisins in human foods such as corn and processed corn-based products and at 5–100 ppm in different animal feeds, which it considers achievable with the use of good agricultural and good manufacturing practices. In 2007, the EU amended the legislation on the maximum levels of fumonisins in maize and maize-based products to 4 ppm in unprocessed maize and 1 ppm in maize intended for direct human consumption.

### 2.5. Trichothecenes

**Figure 5:**
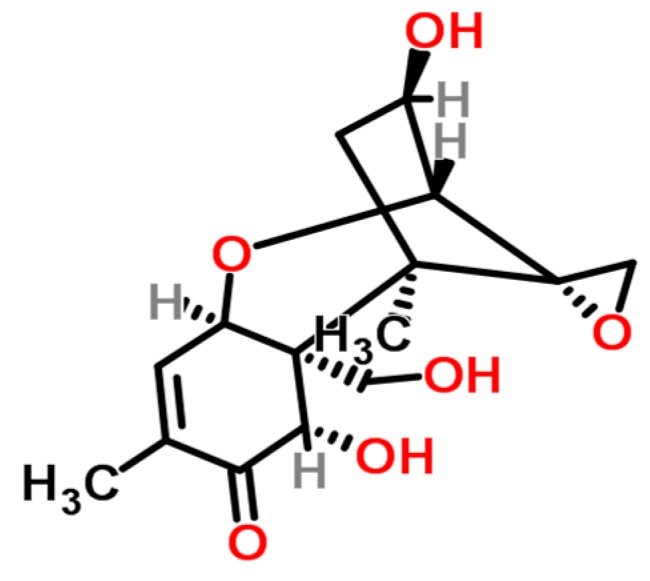
Deoxynivalenol (structure from www.chemspider.com).

Trichothecenes (TCTC) were recognized as causing alimentary toxic aleukia (ATA) toxicosis in the USSR in 1932. Over 150 TCTC variants have been identified to date, but only a few are of agricultural importance [44]. TCTC are the most chemically diversified of all mycotoxins [3]. Among TCTC, deoxynivalenol (DON, Figure 5) is the most common and well-studied, but is also among the least toxic [5].

TCTC are produced mainly by *Fusarium* species fungi. However, *Acremonium (Cephalosporium)*, *Cylindrocarpon*, *Dendrodochium*, *Myrothecium*, *Trichoderma*, *Trichothecium*, and *Stachybotrys* species are also able to produce TCTC [52,53]. *Fusarium* species usually infect and produce TCTC in crop plants in the field [5]. Economically, the most important TCTC producers are *F. graminearum* and *F. culmorum* which cause Fusarium Head Blight (FHB), a destructive disease of cereal grain crops with worldwide economic impact [53]. TCTC mainly contaminate cereals such as wheat, barley, oats, rye, maize, and rice [44]. They may also be present in soybeans, potatoes, sunflower seeds, peanuts, and bananas, and have been found in some processed foods derived from cereals (bread, breakfast cereals, noodles, and beer) [5]. DON is the most widely distributed *Fusarium* mycotoxin, contaminating cereals in Japan, Korea, Europe, Southern Africa, and Australia [54].

The IARC has placed DON in carcinogenesis Group 3 [13]. The oral LD_50_ for DON is 46–78 mg/kg. Human exposure to DON-contaminated grains has been reported to cause nausea, vomiting, diarrhea, abdominal pain, headache, dizziness, and fever [55]. Generally, the common symptoms of TCTC toxicity in animals are slow growth, lowered milk production in cattle, feed refusal, drop in egg production in laying hens, intestinal hemorrhage, and suppression of immune responses [56]. TCTC are highly toxic and can easily penetrate cell membrane lipid bilayers to react with DNA, RNA, and cellular organelles [57]. The major mechanism of TCTC toxicity is inhibition of ribosomal protein synthesis, which is followed by secondary disruption of DNA and RNA synthesis [9]. The US FDA has established advisory levels of 1 ppm DON for finished wheat product such as flour and bran that may be consumed by humans and 5 ~ 10 ppm for all grains and grain by-products intended for animal consumption.

### 2.6. Patulin

**Figure 6:**
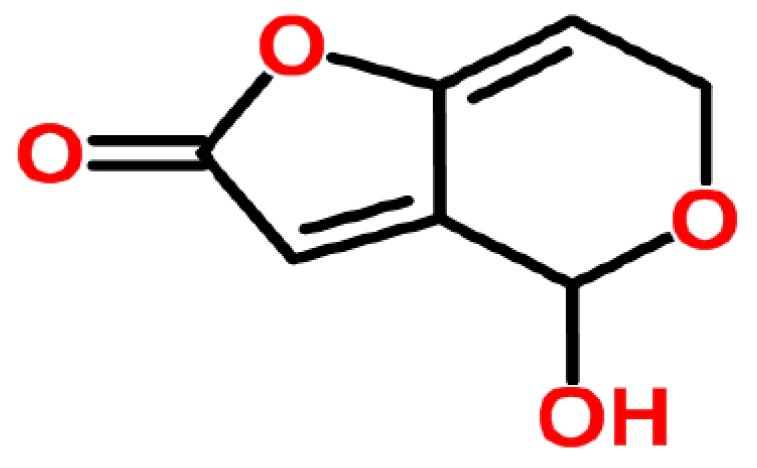
Patulin (structure from www.chemspider.com).

Patulin (Figure 6) is a polyketide mycotoxin discovered in 1943. It is produced by certain species of *Penicillium*, *Aspergillus*, and *Byssochlamys* growing on fruit and vegetables, with *P. expansum* recognized as the most fungus for its production [5,58]. While it predominantly contaminates apples, apple juice, and apple products, other fruit including pears, peach, and grapes may also be vulnerable to patulin contamination [59,60,61]. Patulin was initially studied as a potential antibiotic, but subsequent research demonstrated human toxicities, including nausea, vomiting, ulceration and hemorrhage [62]. In rodents, the oral LD_50_ of patulin ranges from 29–55 mg/kg body weight [63]. Although IARC has expressed much concern about the possible carcinogenicity of patulin, it nevertheless placed patulin in carcinogenicity Group 3 ([64]). The US FDA limits patulin to 50 ppb as an action level in food for human consumption. EU committee has set a maximum level of 50 ppb for fruit juices and concentrated fruit juices, 25 ppb for solid apple products, and 10 ppb for juices and foods consumed by babies and infants.

## 3. Analysis of Mycotoxins in Food

**Figure 7:**
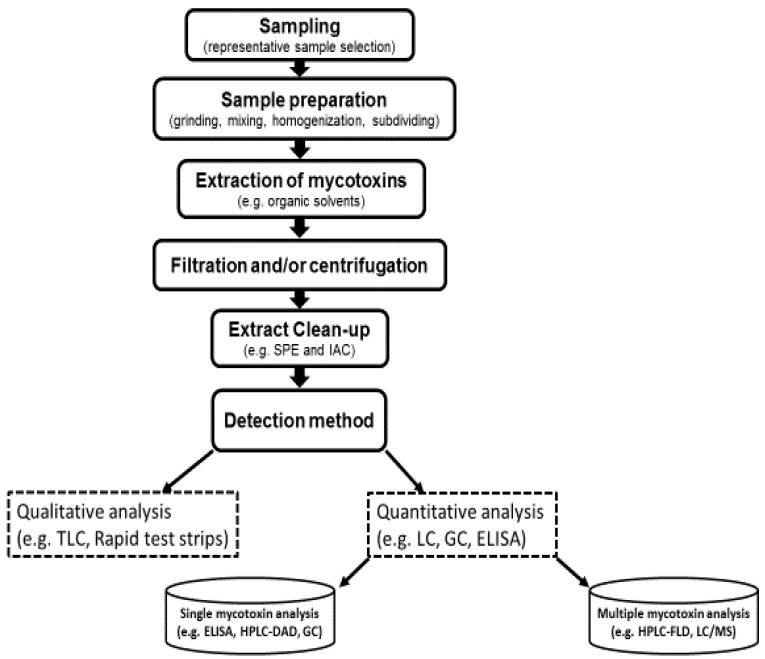
Flow diagram of common steps involved in mycotoxins analysis in food commodities.

A major global food safety issue is the presence of mycotoxins in food products [25,65,66]. Determination of mycotoxin levels in food samples is usually accomplished by methods that include certain common steps: sampling, homogenization, extraction followed by a cleanup, and finally the detection and quantitation which is performed by many instrumental and non-instrumental techniques (Figure 7) [7,19,67].

### 3.1. Sampling Tactics

A key step in the analysis of mycotoxins in food is the sampling procedure, which greatly contributes to the reliability of the results and the final decision of compliance or non-compliance for an entire food batch [17,68]. With the exception of liquid food samples such as milk or some highly processed food (i.e., peanut butter), traditional sampling methods for foodstuffs are usually not suitable for mycotoxins analyses since mycotoxins are not present homogeneously in food [19,69,70]. Due to the uneven distribution of the mycotoxins in food, it is very challenging to get a representative sample of the bulk [70,71]. Thus, a carefully considered sampling plan must be implemented to ensure that the tested sample is representative of the whole bulk and to guarantee the trueness of the results [19]. To address the problems associated with sampling for mycotoxins analysis, many sampling plans have been developed and employed based on statistical parameters that balance consumer safety with producer protection [72,73]. Such sampling methods are described by the EU under the Commission Regulation (EC) No. 401/2006 [16,19]. Nevertheless, continuing efforts are directed towards improving the sampling plan for the analysis of mycotoxins in food and feed which are governed by governmental regulatory agencies worldwide to reduce the variability of the analytical results.

### 3.2. Sample Preparation: Mycotoxin Extraction and Clean-Up

At present, a vast majority of published methods on mycotoxins analysis in food requires intensive sample preparation to separate the toxins from the food matrix [16,73]. Extraction of mycotoxins from solid food samples into a liquid phase is the first step in sample preparation, followed by cleanup procedures to enhance the sensitivity and specificity of a given detection method [74]. The selection of methods for extraction and cleanup of mycotoxins from food samples is usually governed by three major factors: the chemical properties of the mycotoxins, the nature of food matrix, and the detection method that will be used [70].

Most liquid food samples such as milk, wine, and apple juice are subjected to liquid-liquid extraction to initially separate the mycotoxins. However, solid-liquid extraction may also be used, especially for mycotoxin extraction from grains, cereal foodstuffs, and other solid materials [7]. Most mycotoxins are highly soluble in organic solvents such as methanol, acetonitrile, acetone, chloroform, dichloromethane, or ethyl acetate, but hardly soluble in water [70,75,76]. However, as mentioned, fumonisins are soluble in water as they contain four free carboxyl groups and one amino group, and FB1 is highly stable in a mixture of water and acetonitrile [5]. A mixture of organic solvents with the addition of certain amount of water or acidic buffer is frequently used to extract mycotoxins [74,75]. While the addition of water would enhance the penetration of the organic solvents in the food matrix, an acidic solvent can break the strong bonds between the analyte and other food components such as protein and sugars, leading to enhanced extraction efficiency [75]. For samples with high lipid content, non-polar solvents such as hexane and cyclohexane are used [76]. Recently, many instrumental automated solvent extraction methods have been used in mycotoxin analysis, including supercritical fluid extraction (SFE), accelerated solvent extraction (ASE), and microwave-assisted extraction (MAE) [7,77]. Compared to the conventional methods, these methods accelerate mycotoxin extraction, require smaller volumes of chemical solvent (which is therefore more environmentally friendly), and usually provide better extraction efficiencies; however, such automated methods may be costly [78]. After mycotoxin extraction, filtration and centrifugation are important steps to remove any interfering particles before performing further clean-up steps.

Cleanup of the extract is an important process to eliminate those substances that may interfere with the subsequent detection of mycotoxins. By cleaning up the extract, the specificity and sensitivity is enhanced resulting in improved accuracy and precision [76]. A variety of cleanup methods have been implemented including liquid–liquid partitioning, solid phase extraction (SPE), immune-affinity columns (IAC), column chromatography, ion-exchange columns, and multifunctional cleanup columns such as Mycosep™ [7,75]. The most commonly used methods for mycotoxins clean-up are SPE and IAC, as these are rapid, efficient, reproducible, and safe, with a wide range of selectivity [79,80]. SPE is a technique based on the specific partitioning of the analyte dissolved in the extract (mobile phase) and the stationary phase (cartridge), which is composed of a solid adsorbent where the mycotoxins are absorbed and then eluted with an organic solvent [75]. There is a wide range of commercially available column packings with different sorbents such as ethyl, octyl, octadecyl, cyclohexyl, phenyl, cyanopropyl, and aminopropyl functional groups; different sorbents may be used based on the food matrix, the chemical nature of mycotoxins, and the solvent to be used [7,81]. IAC are packed with activated solid phase bound to a specific antibody for a given mycotoxin(s). When the extract passes through the column, the mycotoxin binds selectively to the antibodies, while other matrix component will be removed by a washing step. The mycotoxin is then eluted with a miscible solvent such as methanol [7].

Recently, the Quick, Easy, Cheap, Effective, Rugged, and Safe (QuEChERS) sample preparation method has been applied for extraction and clean-up of mycotoxins from different food matrices [82]. This technique was initially developed in 2003 for pesticide analysis, then adapted to extract a wide range of matrices and analytes such as acrylamide, aromatic amines, polycyclic aromatic hydrocarbons (PAHs), and mycotoxins [83]. The technique involves a simple two-step based solvent extraction, such as acetonitrile in the presence of salts (magnesium sulfate and sodium chloride), and dispersive-SPE (d-SPE) for clean-up [84]. While magnesium sulfate is usually used to remove water from organic phase in the sample, sodium chloride is used to reduce the amount of polar interferences. For the cleanup step, a primary secondary amine (PSA) (e.g., Florisil, alumina, or silica) or C18 is usually used to remove the sugars and lipids, organic acids, and some pigments [70]. QuEChERS is a fast, inexpensive, and simple method that uses minimal amounts of solvent compared to other methods [7,19]. In the last years, this technique has been used for the analysis of multiple mycotoxins in many food matrices such as grain and cereals products; animal by-products such as egg and milk; wine, coffee, and spices; and in the multi-residue extraction of different contaminants (including mycotoxins) in foods [85,86,87,88,89].

### 3.3. Analytical Methods

#### 3.3.1. Chromatographic Techniques

Chromatography is the most commonly used method used for mycotoxin analysis in food and feed [19]. The earliest chromatographic method is thin layer chromatography (TLC), which is presently used as a rapid screening method for certain mycotoxins by visual assessment or instrumental densitometry [73]. However, current trends in mycotoxin analysis in food are focused on application of robust, fast, easy to use, and cheap technologies that are able to detect and quantify various mycotoxins with a high sensitivity and selectivity in a single run [75]. To meet those needs, many chromatographic methods such as high performance liquid chromatography (HPLC) coupled with ultraviolet (UV), diode array (DAD), fluorescence (FLD), or mass spectrometry (MS) detectors and UHPLC or UPLC with reduced column packing material (1–2 μm) have been developed [7]. Additionally, gas chromatography (GC) coupled with electron capture (ECD), flame ionization (FID), or MS detectors have been used to identify and quantitate volatile mycotoxins such as TCTC and patulin [7]. Due to the low volatility and high polarity of most mycotoxins, GC analysis often requires a derivatization step; therefore, this method is used rarely in mycotoxins analysis [90]. Mycotoxin analysis has been greatly advanced by coupling liquid chromatography techniques to mass-spectrometry (e.g., LC-MS; LC-MS/MS) [19]. While HPLC coupled with mass spectrometric or fluorescence detectors are routinely used for analysis of mycotoxins in food, other chromatographic techniques are seldom used due to the limited sensitivity and specificity [7,91].

Among all non-MS chromatographic techniques, HPLC-FLD coupled with an efficient extraction and cleanup method is frequently used for quantitative analysis of mycotoxins, particularly AFs [7,19]. HPLC-FLD methods have been adapted by the Association of Official Analytical Chemists (AOAC) International and by the European Standardization Committee (CEN) for quantification of mycotoxins in cereals [92]. By this technique, it is possible to obtain sensitivity that is comparable to those achieved by LC-MS/MS; however, HPLC-FLD methods are usually most suitable for single mycotoxins or a group of chemically related mycotoxins [93,94]. Recently, a HPLC-FLD method has been employed for the simultaneous detection of multiple mycotoxins: (1) AFs and OTA in maize cereal products, peanut butter, ginseng and ginger [95,96]; (2) AFs, OTA, and ZEA in cereal grains, rye and rice [97,98]; (3) AFs, OTA, ZEA and DON in corn [99]. Although these HPLC-FLD detection methods have relatively good sensitivity and recovery, the requirement for extensive cleanup and pre-/post-column derivatization for proper detection of mycotoxins are downsides.

Apart from the great advantages of the conventional HPLC methods mentioned above, MS offers several distinct advantages over all LC methods for mycotoxin analysis in food. Basically, the mass spectrometer works by ionizing the molecules, and sort and identify them based on their mass-to-charge ratio (*m/z*) [79]. MS offer higher sensitivity and selectivity, as well as chemical structural information by molecular identity of the analyte based on *m/z* providing the mass spectrum as an ideal confirmatory technique [100,101,102]. MS detection reduces time by eliminating the need for error-prone sample derivatization and cleanup steps needed for fluorescence enhancement [19]. Different MS interfaces and analyzers have been used, such as atmospheric pressure chemical ionization (APCI), electrospray ionization (ESI), and atmospheric pressure photo-ionization (APPI) [7]. In addition, there are many types of mass analyzers such as quadrupole, time-of-flight (TOF), ion-trap, and Fourier transform-ion cyclotron resonance (FT-ICR). ESI, triple quadrupole, and TOF have been used extensively for mycotoxin analysis [16,76]. Although the early applications of MS were for the analysis of single mycotoxins, the technique can simultaneously quantify over 100 mycotoxins in a single run, making it the current method of choice for detecting multiple mycotoxins in a wide variety of foods, and [79].

#### 3.3.2. Immunochemical Methods

Among all published immunological based methods, the enzyme-linked immunosorbent assay (ELISA) is probably most commonly used for mycotoxin determination. ELISA provides rapid screening, with many kits commercially available for detection and quantification of all major mycotoxins including AFs, AFM1, OTA, ZEA, DON, fumonisins, and T-2 toxin. ELISA methods have been validated in a wide variety of food matrices [7,75]. While ELISA can be performed in several ways such as direct assay, competitive direct assay, and competitive indirect assay, a competitive direct assay is most commonly used [103,104]. The principle of ELISA is based on the competitive interactions between mycotoxins (acting as an antigen) and assigned antibodies labelled with toxin-enzyme conjugate for many binding sites [76,105]. The amount of antibody-bound toxin-enzyme conjugate will determine the level of color development [106]. This technique provides a rapid, specific, and relatively easy to use method for analysis of mycotoxins in food. However, ELISA has certain disadvantages including potential cross-reactivity and dependence on a specific matrix. In addition, the kit detects only a single mycotoxin and is designed for one-time use; thus, it can be costly if one needs to test samples contaminated with multiple mycotoxins [7,106]. Moreover, each test kit is specified by the manufacturer [105]. While some third-party validations, e.g., by AOAC, have been done for some mycotoxin ELISA kits, the validation and marketing are for use with specific toxins under specific contamination levels within specified matrixes; therefore, the kit cannot be used for all food matrices and all contamination levels [106]. Positive ELISA results should be confirmed by a suitable chromatographic method, especially when used in a matrix not specified by the manufacturer [19].

#### 3.3.3. Rapid Methods

Rapid diagnostic test kits such as the pregnancy and blood sugar test strips have been used for many years in the medical field. In the last decade, there is ongoing interest in developing rapid on-site test strips for detection of major food contaminants such as foodborne pathogens, veterinary drug residues, pesticides, allergens, and mycotoxins [107,108]. These test methods are designed to be performed outside the laboratory at the site of inspection. Results are expected to be obtained within a short time, with the help of simple portable devices or even without using any instrument or readers [109]. Besides the common ELISA procedures, many kinds of rapid visual immunoassay strips for on-site testing of mycotoxins are commercially available, including lateral-flow (LFD), dipstick, and flow-through devices [7,77].

LFD has been developed as a single-step test that includes a negative control line along with the sample lines on the same strip. A lateral flow test can provide semi-quantitative results in less than 10 min and requires no specialized equipment [7]. It consists of three parts: a conjugate pad, a porous membrane, and an absorbent pad [77]. The test is based on a competitive immunoassay, where a labeled antibody is used as a signal reagent [109]. This device has recently been coupled with spectrometric readers to provide quantitative results [107]. LFDs are commercially available for detection of AFs, DON, T-2 toxin, OTA, and ZEA [109]. However, their applications in the field is limited due to numerous problems associated with the sensitivity and reliability in different matrices in addition to their high cost [108].

Dipstick test work similarly to ELISA and require preparation and incubation steps to obtain the results, which usually takes more than 30 min [77]. Dipsticks are commercially available for the detection of single mycotoxin contamination in food. The first dipstick assay was developed for the detection of FB1 in corn-based foods, with a visual limit of detecting 40–60 ng/g [110]. A multi-analyte dipstick immunoassay for the detection of various mycotoxins was next developed, however, its sensitivity is limited. It is also available as a multiplex dipstick immunoassay for the simultaneous detection of major *Fusarium* toxins such as ZEA, T-2, and HT-2 toxins, DON and fumonisins in wheat, oats, and maize [111], DON and ZEA in wheat [112], and ZEA and OTA in maize [107,113].

Flow-through membranes utilize the same basic principle as LFD but may not yield accurate results near the detection limit [107,109]. Flow-through immunoassays have been developed for screening OTA in green and roasted coffee [114,115], AFB1 in nuts [116], and ZEA in cereals and feed samples [117]. Although many different rapid strip tests have been developed for detection of major mycotoxins in different food commodities, they are not commonly used in the field and not commercially successful due to problems related to sensitivity, cost, and accuracy [109].

#### 3.3.4. Other Emerging Detection Technologies

In addition to the methods described above, several other research methods have potential utility for the analysis of mycotoxins. However, these methods have limited application and have not been widely used outside the research environment as they require further verification and validation by recognized bodies such as AOAC, International Organization for Standardization (ISO) or CEN.

*Infrared spectroscopy*: Optical methods the incorporate infrared (IR) analyzers coupled with principal component analysis (PCA) for screening and quantification of mycotoxins without sample preparation are promising fast and non-destructive techniques for mycotoxins detection in cereals [118]. Both near-IR reflectance spectroscopy and mid-IR infrared transmittance spectroscopy have been used for detection of DON contamination in wheat and maize [119,120,121]. Recently, a portable IR laser spectroscopy for on-site analysis of DON and AFB1 in maize, wheat, and peanuts samples has been developed [122]. The advantages of these methods are the ease of operation, requiring no chemicals, sample preparation, or extraction, and their rapid results [118]. However, further work is needed to develop the full potential of IR spectroscopy for detecting different mycotoxins, as both methods face challenges, including the non-homogeneous distribution of mycotoxins within the food matrix, particle size distribution of ground grains, and the detection limits of the method [73].

*Capillary electrophoresis*: Capillary electrophoresis (CE) is an instrumental technique that separates different components based on electrochemical potential using fluorescence or UV absorbance [123]. A distinctive advantage of this technique is the small volumes of solvents and buffers required, thereby generating only small volumes of waste [73]. A number of mycotoxins such as AFs, DON, fumonisins, OTA and ZEA, have been separated by CE [124]. However, this method lacks sensitivity as only small sample volumes can be tested [123]. CE coupled with laser-based fluorescence detection, however, has enhanced sensitivity for analysis of FB1, AFs, and OTA in maize, coffee, and sorghum, at efficiencies parallel to those achieved by some chromatographic techniques [125]. Recently, CE coupled with cyclodextrin-enhanced fluorescence has been used for analysis of ZEA in maize with a detection limit of 5 ng/g [124].

*Molecular imprinting polymers*: Molecular imprinting polymers (MIPs) is a synthetic technique that is designed to mimic natural recognition entities such as antibodies and biological receptors with specificities comparable to those of antibody-antigen interactions [126]. The technology utilizes cross-linked polymers that are electrochemically synthetized by the reaction of monomer and cross linker in the presence of an analyte, such as mycotoxins [127]. The main advantages of MIPs are their high selectivity and affinity for the target molecule used in the imprinting procedure, their chemical stability, the ease of preparation, and low cost [128]. MIPs have been designed for the analysis of AFs [127], OTA [129,130], DON, and ZEA [131,132]. While its applicability to food matrices has yet to be demonstrated, and validated, this technique offers excellent potential for further development [133].

*Biosensors*: Biosensors have received considerable attention in recent years as rapid, reliable, and low-cost tools for quantification of mycotoxins in foodstuffs [134]. A biosensor is a measuring device that incorporates a specific biological element (e.g., antibody) that creates a biorecognition event and a physiochemical element that transduces the recognition event into a electrochemical, optical, piezoelectric, or thermal signal [135]. Various biosensor formats have been developed and used for detection of mycotoxins, such as surface plasmon resonance, fiber-optic probes, and array biosensors [135]. Competitive surface plasmon biosensors have been used for rapid screening for AFB1, ZEA, OTA, FB1, and DON in naturally contaminated matrices [135,136,137]. One major advantage of biosensors is their potential for recycled use, which distinguishes them from single-use ELISA kits and other rapid screening strip tests. Surface plasmon biosensor chips with immobilized DON can be re-used over 500 times without significant loss of activity [137]. While many biosensor formats have the potential to be effective in mycotoxins analysis, most of the biosensor procedures still require sample cleanup. Moreover, the devices are unable to perform simultaneous analyses of multiple analytes [134].

*Fluorescence polarization*: Fluorescence polarization (FP) is a technique widely used clinically for the diagnosis of certain diseases and monitoring therapeutic drug levels in the body fluids [138]. Recently, this technique has been adopted to mycotoxin analysis. FP is a simple technique that measures the interaction between fluorescently labeled antigen and a specific antibody [139]. Basically, this technique indirectly measures the rate of rotation of a fluorophore in solution, which is directly related to the size of a molecule. FP therefore allows detection of low molecular weight materials in solution without requiring a step to separate the toxin [139]. FP have been used for rapid determination of AFs [140], OTA [141], DON [142], fumonisins [143], and ZEA [144]. However, the technique has limited sensitivity and accuracy compared to HPLC, likely due to antibody cross-reactivity towards other fungal metabolites and possibly from food matrix componentnets [145].

*Electronic nose*: An electronic nose (EN) is a variant of GC that mimics the human olfactory sensory system and provides non-destructive, rapid, and low-cost analysis of mycotoxins in food samples [146]. It consists of an array of chemical sensors with different specificities that interact with different volatile compounds. These interactions generate signals that can be utilized effectively as a fingerprint of the volatile molecules rising from the analyzed samples [147]. After achieving a fingerprint, identifying and quantifying odors by means of pattern recognition system can be done [148]. Current applications of EN for mycotoxin detection have focused on the detection of toxigenic fungi rather than detecting the mycotoxin itself. This technique is practically useful for differentiating between toxigenic and non-toxigenic fungi [146,149], and has been used to discriminate between moldy and non-moldy grains [150]. A few studies have been published on the using of EN for AF detection in corn [151] and DON in grain [149]. Using this technology for analysis of mycotoxins in food is still in the early development phase. Instrumentation needs to be optimized for the quantification of low levels of mycotoxins in food samples. Additionally, most of mycotoxins are non-volatile organic compounds, which pose a problem for EN-based detection.

## 4. Conclusions 

Mycotoxins are unpredictable and unavoidable contaminants in foods and feeds worldwide. These small chemicals represent an open challenge for food safety and pose a serious risk for human and animal health while also contributing to massive economic losses to the agriculture industry. Tremendous efforts have been made to control or minimize mycotoxin occurrence in food both in the US and worldwide, but mycotoxin contamination of foods remains problematic. In order to minimize human and animal exposure to mycotoxins, various sensitive and accurate analytical methods have been developed. While HPLC-FLD method is preferred for single mycotoxin analysis, HPLC-MS/MS is the method of choice for simultaneous determination of multiple mycotoxins. Various immunological assays such as ELISA and other rapid antibody-based strip test kits are commercially available for screening mycotoxins in different food commodities on-site or in the laboratory. Moreover, many other promising novel techniques have been proposed for analysis of mycotoxins in food, but these methods require further validation. Collaborative and continuous efforts of governmental authorities, academia, and industry is needed to control mycotoxin production in field, inhibit growth of toxigenic molds in food and feed, and improve detection techniques in order to enhance food safety.

## Figures and Tables

**Table 1:** Major mycotoxins and US and EU limits on food and animal feed levels.

Mycotoxin	Fungal Species	Food Commodity	US FDA (µg/kg)	EU (EC 2006) (µg/kg)
Aflatoxins B1, B2, G1, G2	*Aspergillus flavus Aspergillus parasiticus*	Maize, wheat, rice, peanut, sorghum, pistachio, almond, ground nuts, tree nuts, figs, cottonseed, spices	20 for total	2–12 for B1 4–15 for total
Aflatoxin M1	Metabolite of aflatoxin B1	Milk, milk Products	0.5	0.05 in milk 0.025 in infant formulae and infant milk
Ochratoxin A	*Aspergillus ochraceus* *Penicillium verrucosum* *Aspergillus carbonarius*	Cereals, dried vine fruit, wine, grapes, coffee, cocoa, cheese	Not set	2–10
Fumonisins B1, B2, B3	*Fusarium verticillioides* *Fusarium proliferatum*	Maize, maize, products, sorghum, asparagus	2000–4000	200–1000
Zearalenone	*Fusarium graminearum* *Fusarium culmorum*	Cereals, cereal products, maize, wheat, barley	Not set	20–100
Deoxynivalenol	*Fusarium graminearum* *Fusarium culmorum*	Cereals, cereal products	1000	200–50
Patulin	*Penicillium expansum*	Apples, apple juice, and concentrate	50	10–50
